# Silent struggles: help-seeking barriers for sexual difficulties among adults 50+ in Czechia

**DOI:** 10.3389/fpsyg.2025.1622872

**Published:** 2025-08-05

**Authors:** Gabriela Gore-Gorszewska, Anna Ševčíková, Klára Bártová, Lucie Krejčová, Lucie Kalenská, Renáta Androvičová, Petr Weiss, Kateřina Klapilová

**Affiliations:** ^1^Psychology Research Institute, Faculty of Social Studies, Masaryk University, Brno, Czechia; ^2^Institute of Psychology, Department of Philosophy, Jagiellonian University, Krakow, Poland; ^3^Center for Sexual Health and Interventions, National Institute of Mental Health, Klecany, Czechia; ^4^Faculty of Humanities, Charles University, Prague, Czechia; ^5^Institute for Sexology, General Faculty Hospital, Prague, Czechia

**Keywords:** sexual difficulties, help-seeking behavior, distress, older adults, adults 50+, barriers to help seeking, national survey data, CzechSex

## Abstract

Although sexual problems become more common with age, older adults rarely seek professional help. Understanding why is key to supporting sexual health in aging populations. This study assessed the prevalence of sexual difficulties, help-seeking behavior, and reasons for not seeking help among Czech adults aged 50–75, using nationally representative data from the 2023/2024 CzechSex survey (*n* = 2,927; 53% men). Logistic regression analyses examined the predictor role of sociodemographic factors, sexual activity frequency, sexual ageism, and distress over sexual problems on help-seeking. Lifetime sexual difficulties were reported by 59% of respondents, and 31% experienced them in the past 12 months. Among those with persistent issues, only 7.6% sought counseling or other professional help. Women were significantly more likely than men not to seek help (OR = 1.64, 95% CI [1.04, 2.61], *p* < 0.05); lower distress was also associated with non-help-seeking (OR = 0.68, 95% CI [0.54, 0.86], *p* < 0.001). The most common reasons for not seeking help were perceiving problems as not bothersome, followed by shame, embarrassment, and difficulty communicating (personal/emotional barriers). Systemic barriers (e.g., lack of services, long wait times) were rarely reported. Overall, help-seeking for sexual problems is uncommon in this age group, and personal barriers outweigh institutional ones, posing a challenge for effectively targeting no-help seekers and designing effective interventions.

## Introduction

Sexual activity remains an important aspect of life well into midlife and older age, with abundant evidence that many individuals maintain interest and engage in partnered and solo sexual behaviors (Freak-Poli et al., 2017; Lee et al., 2016; Smith et al., 2019; Træen et al., 2019). For aging adults, sexual engagement and intimacy are linked to wellbeing and relationship satisfaction (Træen et al., 2019). However, sex-related difficulties become more common with age and can hinder sexual activity (Briken et al., 2020; Burghardt et al., 2020; Hendrickx et al., 2015; Richters et al., 2022). Despite this, most older adults do not seek help, and the reasons behind this reluctance remain poorly understood. Existing evidence is limited, drawn mainly from surveys in high-income Western countries or small-scale qualitative studies (Teo et al., 2022). To address this gap, we used a nationally representative sample of Czech adults aged 50–75 to assess the prevalence of sexual difficulties and examine the correlates of and reasons for not seeking help. Czechia provides a unique context as a highly secular, post-communist country (Hamplová, 2013), potentially shaping societal attitudes toward sexuality and willingness to seek professional support.

While it is well established that both the frequency of sexual activity and sexual function decline with age (Lee et al., 2016; Mitchell et al., 2015; Schick et al., 2010), recent population-based studies consistently show that sexual difficulties increase with age, with a notable proportion of midlife and older adults reporting sexual problems (Mitchell et al., 2015; Graham et al., 2020; Hald et al., 2019; Hendrickx et al., 2015; Quinn-Nilas et al., 2018). In the UK Natsal-3 survey, 50.3% of sexually active individuals aged 45–74 reported at least one sexual difficulty in the past year (Mitchell et al., 2013). Similarly, 42.1% of Australians aged 50–69 (Richters et al., 2022) and around 24% of Germans aged 46–75 (Briken et al., 2020) reported such issues. Notably, distress levels are substantially lower most older adults experiencing sexual difficulties report minimal associated concern or distress (Briken et al., 2020; Fischer and Træen, 2021; Graham et al., 2020; Hald et al., 2019; Richters et al., 2022), suggesting a dissociation between dysfunction and perceived impairment. Some studies even suggest that late-midlife and older individuals may downplay these problems (Gore-Gorszewska, 2020; Ševčíková et al., 2023). Nevertheless, sexual difficulties remain an important public health concern requiring recognition and support.

Despite many sexual difficulties being amenable to medical or psychological treatment (Frühauf et al., 2013), help-seeking remains infrequent across all age groups, including among those reporting distress (Hobbs et al., 2019). In a cross-national study, only about 20% of adults aged 40–80 sought professional help for sexual problems (Moreira et al., 2005), and just 10.7% of Britons aged 55–74 sought advice in the past year (Mitchell et al., 2013). More recently, only 12% of men and 7% of women aged 60–75 in four European countries had sought help within five years (Hinchliff et al., 2020). In the U.S., only 17.3% of community-dwelling adults aged 65–80 discussed sexual health with a healthcare provider within two years (Agochukwu-Mmonu et al., 2021). Some surveys lack reporting of help-seeking behaviors alongside sexual problem prevalence, or do not disaggregate by age group, limiting insights into older adults' behaviors (Briken et al., 2020; Richters et al., 2022).

Qualitative studies suggest that barriers to help-seeking among older adults include not being bothered by the symptoms, normalization of symptoms with aging, expectations of symptom resolution without intervention, embarrassment, and concerns about healthcare providers' attitudes and expertise (Bauer et al., 2016; Fileborn et al., 2017; Gore-Gorszewska, 2020; Hinchliff et al., 2020; Schaller et al., 2020; Teo et al., 2022).

Unaddressed sexual problems in later life can negatively impact personal and relational satisfaction, and sexual and general wellbeing (Lodge and Umberson, 2012; Ševčíková et al., 2023). Understanding why aging individuals often do not seek help for these issues is therefore essential. This study aimed at examining the extent to which Czech adults aged 50–75 seek professional help for sexual difficulties, the characteristics of those who report sexual difficulties but do not seek support, and finally, the reasons behind the lack of help-seeking.

## Materials and methods

### Procedure

The study was part of CzechSex, a nationwide survey of sexual wellbeing and behavior conducted in Czechia from December 2023 to March 2024. The methodology followed best practices for sexual health surveys and employed a combination of methods that allow for cost-efficient balance by using face-to-face methods (Computer-Assisted Self-Interviewing [CASI], Computer-Assisted Personal Interviewing [CAPI]) for hard-to-reach populations and online methods (Computer-Assisted Web Interviewing [CAWI]) for broader outreach.

### Sampling

Data were collected using quota sampling based on gender, age, education, region, and residence size, reflecting the Czech population structure from the 2021 census (Czech Statistical Office, 2023). Response rates were 46.98% for CAPI/CASI and 28.39% for CAWI.

### Participants

Participants included 6,669 Czech adults aged 18–75, with 2,009 interviews conducted in person and 4,660 online. This study uses a sub-sample of 2,939 adults aged 50–75 (Mean age = 61.92, SD = 7.52); with 1521 women (51.8%). Most of the respondents self-identified as heterosexual (97.5%), were married or in a civil partnership (56.8%) and still work-active (55.3%) (see Table 1 for demographic details, weighted data).

**Table 1:** Sociodemographic characteristic and descriptive statistics of the sample (weighted data).

**Descriptives**	**Respondents aged 50**+ **(*****n*** = **2939)**		**Having sexual problems (*****n*** = **1733)**		**Having sexual problems in the past 12 months (*****n*** = **919)**	
	**rate/mean**	**SD**	**rate/mean**	**SD**	**rate/mean**	**SD**
**Sex**						
Men	48.20		47.72		47.77	
Women	51.76		52.28		52.23	
**Age**						
50–59	41.88		38.72		38.30	
60+	58.12		61.28		61.70	
**Sexual orientation**						
Heterosexual	97.50%		97.21%		97.10%	
Non-heterosexual	2.50%		2.79%		2.90%	
**Education**						
Primary/Vocational	44.38%		44.20%		46.79%	
Secondary	35.88%		35.39%		33.08%	
Tertiary	19.74%		20.31%		20.13%	
**Employment status**						
Employed	55.30%		53.01%		51.25%	
Unemployed/Work inactive	4.54%		5.19%		5.01%	
Retired	40.16%		41.78%		43.74%	
**Religiosity**						
Religious affiliation	28.40%		29.62%		29.21%	
No religious affiliation	71.60%		70.38%		70.38%	
**Marital status**						
Single	7.14%		6.75%		6.86%	
Married/Civil partnership	56.77%		56.03%		57.19%	
Divorced/Dissolution	24.26%		25.68%		23.31%	
Widowed	11.83%		11.54%		12.64%	
**Relationship status**						
In a committed relationship	73.11%		72.73%		72.11%	
No relationship	26.89%		27.27%		27.89%	
**Other descriptives**						
Relationship length	20.67	18.16	20.67	18.34	21.41	18.74
Self-perceived health in past 4 weeks	2.55	0.89	2.68	0.91	2.83	0.91
Sexual frequency in past 12 months	2.93	1.10	2.21	1.10	2.09	1.06
Sexual ageism	1.60	0.97	1.50	0.86	1.48	0.84
Sexual problems in the past 12 months			59.29%			
Level of distress over sexual problems			2.71	0.94	2.84	0.97
**Seeking help for sexual problems**						
No help-seeking behavior			92.35%		89.26%	
Seeking help			7.64%		10.74%	

### Ethics

Responses were anonymous and confidential. The study was approved by the National Institute of Mental Health's Institutional Review Board (Approval No. 119/19) participants were informed about the study's purpose and data handling.

### Measurements

#### Sexual problems

After the introductory sentences which normalized the existence of problems related to sexual function and varied levels of distress, a question with eight statements about experiencing listed sexual problems was provided: “Have you ever had any of these problems, for at least a few months?” (e.g., “My sex drive was greatly reduced, or I had none”, “I have not been able to achieve or maintain an erection, or it was not firm enough to be sexually active”, “I have experienced intense and persistent or recurrent difficulties during sexual intercourse, e.g., due to pelvic floor muscle spasms, pain or fear of pain.”). These statements were adapted from the GeSiD survey (Briken et al., 2020).

The response options were Yes (=1), No (=0), and “It does not concern me,” coded as a missing value. Respondents were also asked whether they had experienced these sexual problems in the past 12 months, with response options Yes (=1) and No (=0).

#### Help-seeking behavior

Participants who reported experiencing at least one sexual problem in the past were asked “Have you sought counseling or other professional help for these sexual problems in the last 5 years?” The response options were Yes and No.

*Reasons for not seeking help* were assessed by the question “Why didn't you seek counseling or professional help? Tick all that apply”, administered to all respondents who reported no help-seeking. The alternatives were “I was ashamed, I was embarrassed” (=1), “It was hard for me to talk about my problem” (=2), “I didn't want anyone to know about my problem” (=3), “I didn't know where to turn for help” (=4), “Waiting times were too long” (=5), “No such services were offered where I live” (=6), “The last time I sought help, I had negative experience or was disappointed” (=7), “I didn't have time to deal with it” (=8), “I assumed it would work itself out” (=9), “The symptoms didn't bother me much” (=10), “My partner didn't agree with that” (=11), “Other reasons” (=12). Response options 1–4 were grouped into the category “Personal/Emotional barriers”, and response options 5–7 formed the category “Systemic/Institutional barriers”.

*Self-assessed general health status* was measured by the question “In general, how do you rate your physical health in the last 4 weeks:” where the response categories were 1 = very good, 2 = good, 3 = average, 4 = poor, and 5 = very poor.

*Length of current relationship*. Respondents were asked to estimate relationship duration in years or months (if shorter than 1 year).

The predictors of the outcome variables above were *Age* and *Sex* (1 = male, 2 = female). Although non-heterosexual individuals were included in the overall sample, their small number did not allow for reliable analysis, they were therefore excluded from the regression models. *Level of education* was assessed based on the highest level of formal education, with response options including primary or vocational (=1), secondary education with a diploma (=2), higher or tertiary education (=3). *Religiosity* was assessed with a single question on religious affiliation, with response options including Christianity, Islam, Judaism, Buddhism, Hinduism, Sikhism, Bahá'í, Other, and No religious affiliation. For analysis, the item was dichotomized as No religious affiliation (=0) and Religious affiliation (=1). *Relationship status* was assessed by a question “Are you currently in a stable/committed relationship? This could include romantic relationships, marriage, or partnerships, regardless of length, cohabitation, or sexual activity”. Response options (Yes, with a woman/a man/a trans, non-binary, or gender-diverse person/multiple partners of the same gender/multiple partners of different genders; No, I do not have a stable/committed partnered relationship) were dichotomized as being (=1) and not being in a committed relationship (=0). *Frequency of sexual activity* was measured with a single item: “Approximately how often have you had sex in the last 12 months? By sex we mean any sexual contact between people, involving the genital area. This may include oral sex, vaginal sex, anal sex, fondling/touching, genital rubbing, etc.”. Respondents indicated the frequency on a 6-point Likert scale ranging from not once (=1) to several times per day (=6). A higher score indicated more frequent sexual activity. *Sexual ageism* was measured by a single item: Respondents rated their attitudes toward older adults engaging in an active sexual life on a 5-point Likert scale, ranging from completely acceptable (1) to completely unacceptable (5), with higher scores indicating stronger sexual ageism. *Level of distress*: Items assessing how distressing a sexual problem was were shown only to participants who reported experiencing that specific problem. All displayed items, which used a 5-point Likert scale, ranging from not at all (=1) to very strongly (=5), were averaged. A higher score indicated the problems were perceived as more distressing.

#### Analyses

Apart from the descriptive data analysis, we conducted logistic regression to explore correlates of seeking professional help for sexual problems. Based on sensitivity analysis conducted in G^*^Power (Faul et al., 2009), by conducting a logistic regression analysis with a sample size of at least 844 people, the alpha level set to 0.05, and statistical power of 0.80, it was expected to detect the medium effects of R2 increase = 0.05 (OR = 1.25) and higher. The outcome variable was seeking (=0) and not seeking counseling or other professional help for sexual problems (=1). Sex, age group (50–59/60+), education, religiosity, relationship status, frequency of sexual activity, sexual ageism, and the level of distress over experienced sexual problems were included as independent variables. The assumptions of the logistic regression were satisfied. There was no significant multicollinearity (VIF < 3) in any the tested model. The analysis was carried out using weighted data in the statistical package SPSS 24.

## Results

Table 1 provides characteristics of the whole sample and the sample of those who indicated having any sexual problems in their life and in the past 12 months. About 59% of the sample had a past experience with sexual problems lasting at least several months. There were no sex differences [χ^2^(1) = 2.817], 52.28% of women and 47.72% of men reported experiencing at least one sexual problem. However, there were significant age differences [χ^2^(1) = 18.992^***^], 38.72% of people aged 50–59 and 61% of people aged 60–75 reported experiencing at least one sexual problem. About 53% of those who had experienced at least one sexual problem had dealt with it in the past 12 months. Of these, 44.7% indicated that the sexual problems they experienced in the past 12 months were somewhat distressing, while 18.9% found their difficulties to be very distressing. Among those who were sexually active in the past 12 months (*n* = 1,803), 29.4% reported experiencing sexual difficulties during that time.

### Frequency of professional help-seeking

Only a fraction of participants (7.6%, *n* = 130) had sought counseling or professional help for a sexual problem in the past five years. More men than women reported seeking help [χ^2^(1) = 10.763^***^]: 9.8% of men reporting at least one sexual problem (*n* = 80) and 5.6% of women reporting at least one sexual problem (*n* = 50). There were no significant age differences [χ^2^(1) = 0.40] in help-seeking behavior: 8.2% in the 50–59 group (*n* = 54) and 7.5% in the 60–75 group sought counseling or professional help in the past five years.

### Characteristics of no-help seekers

We conducted logistic regression to predict who reported experiencing at least one sexual problem in the last 12 months but did not seek professional help (Table 2). The analysis showed that help seekers and no-help seekers did not differ in most sociodemographic characteristics such as age, education level, relationship status, religiosity. Additionally, neither frequency of sexual activity nor sexual ageism predicted seeking professional help. Only sex and level of distress over sexual problems were associated with help-seeking. Women were more likely to not seek counseling or professional help for sexual problems experienced in the past 12 months (OR = 1.64; p < 0.05; 95% CI [1.04; 2.61]). The perception of sexual problems as less distressing was also associated with not seeking professional help (OR = 0.68; *p* < 0.001; 95% CI [0.54; 0.86]).

**Table 2:** Not seeking counseling or other professional help for sexual problems experienced in the past 12 months (*n* = 844, weighted data).

**Factors**	**No help seekers**	
	**OR**	**OR 95% CFI**
**Sex**		
Men	1	
Women	1.64^*^	(1.04, 2.61)
**Age**		
50–59	1	
60+	1.20	(0.76, 1.90)
**Education**		
Primary/Vocational	1	
Secondary	1.43	(0.85, 2.41)
Tertiary	1.20	(0.76, 1.90)
**Religiosity**		
No religious affiliation	1	
Religious affiliation	1.08	(0.65, 1.78)
**Relationship status**		
No relationship	1	
In a committed relationship	1.04	(0.59, 1.83)
**Other factors**		
Sexual frequency in the past 12 months	1.86	(0.68, 1.08)
Sexual ageism	1.10	(0.82, 1.48)
Level of distress over sexual problems	0.68^***^	(0.54, 0.86)
**Model fit**		
Hosmer-Lemeshow Test	χ^2^(8) = 9.10	
Nagelkerke's *R*^2^	0.05	

^*^p < 0.05, ^***^p < 0.001.

### Reasons for not seeking professional help

In total, 90.11% (*n* = 729) of men and 94.36% (*n* = 836) of women who had experienced a sexual problem lasting at least several months claimed they did not seek professional help. The most common reason for not seeking professional help, reported by around one third of women and men was not perceiving symptoms as bothersome. Around 1 in 4 individuals, irrespective of sex and age group, claimed they have not sought help due to feeling ashamed, embarrassed or not knowing how to talk about the issue (personal/emotional barriers). Men, especially those aged 50–59, tended to report more than women that they assumed symptoms would resolve on their own as a reason for not seeking professional help. Still, almost 1 in 5 women reported the assumption of self-resolution. The fourth barrier was a lack of time, indicated by < 1 in 10 individuals, slightly more often in adults 50–59 than 60+. Systemic/Institutional barriers (i.e. lack of services, long waiting times, negative past experiences) were reported infrequently, along with partner disagreement and other, not specified reasons (Table 3).

**Table 3:** Reasons for not seeking help in the past five years among respondents who had experienced one or more sexual problem, by gender and age group (weighted data).

**Reasons**	**Men**		**Women**	
	**50–59 (%) (*****n*** = **284)**	**60**+ **(%) (*****n*** = **444)**	**50–59 (%) (*****n*** = **322)**	**60**+ **(%) (*****n*** = **515)**
The symptoms didn't bother me so much	29.8 (85)	31.8 (141)	29.5 (95)	27.2 (140)
Personal/Emotional barriers^a^	25.4 (72)	21.9 (97)	25.8 (83)	24.1 (128)
I assumed it would work itself out	31.7 (90)	25.2 (112)	19.5 (82)	18.1 (93)
I didn't have time to deal with it	9.2 (26)	6.8 (30)	10.9 (35)	6.4 (33)
Systemic/Institutional barriers^b^	3.2 (9)	3.2 (14)	4.7 (15)	4.7 (24)
My partner didn't agree with that	0	1.8 (8)	1.9 (6)	1.9 (10)
Other reasons	7.0 (20)	8.1 (36)	6.8 (22)	6.8 (35)

Multiple answers allowed; thus, percentages do not sum up to 100.

^a^Personal/Emotional barriers: “I was a I was ashamed, I was embarrassed”, “It was hard for me to talk about my problem”, “I didn't want anyone to know about my problem”, “I didn't know where to turn for help”.

^b^Systemic/Institutional barriers: “Waiting times were too long”, “No such services were offered where I live”, “The last time I sought help, I had negative experience or was disappointed”.

## Discussion

Using representative data, this study aimed at examining the extent to which Czech adults aged 50+ seek counseling or other professional help for sexual difficulties, the characteristics of those who report sexual difficulties but do not seek help, and the reasons behind the lack of help-seeking behavior.

The present study showed that nearly 59% of Czechs aged 50–75 have experienced sexual problems in their lifetime, while 31% reported such difficulties in the past 12 months. Among those who were sexually active, only 29.4% reported experiencing sexual difficulties in the past 12 months—considerably lower than in other population-based surveys (Mitchell et al., 2013; Richters et al., 2022). However, these rates appear to be more comparable to the GeSiD (24% of people aged 46–75 reported experiencing sexual problems in the previous 12 months; Briken et al., 2020), probably due to methodological similarities in measuring sexual difficulties used in both surveys.

The rates of help-seeking in Czechia confirm patterns observed in other population-based studies that provide age-specific data (Hinchliff et al., 2020; Mitchell et al., 2013; Moreira et al., 2005). Only a small proportion (7.6%) of Czech people aged 50–75 had sought counseling or professional help for a sexual problem in the past five years. This echoes recent results from a representative survey in four European countries, where 9.45% of individuals aged 60–75 had sought professional help for a sexual problem in the same time frame (Hinchliff et al., 2020), and is slightly lower than in the Natsal-3 project, where 10.7% of British people aged 55–74 reported seeking help or advice regarding their sex life from any source (e.g., a friend, the internet, or professional services) in the past year (Mitchell et al., 2013). It is worth noting that, unlike the Natsal-3 data, the CzechSex survey focused exclusively on counseling or professional help—a specific form of help-seeking that may be less common than seeking sexually related advice from informal sources or online. Overall, this indicates that Czechs, akin to their peers elsewhere, rarely seek professional help for sexual difficulties despite the relatively high rates of in-person consultations with medical doctors reported by Czechs (Eurostat, 2024). This poses a broader challenge for improving sexual health and wellbeing among aging populations in Czechia and beyond.

### Correlates of help-seeking

Our logistic regression analysis confirmed that the level of distress over sexual problems was a key factor in seeking professional help in Czech adults 50+. This aligns with prior research identifying similar patterns (Hinchliff et al., 2020; Moreira et al., 2005; Štulhofer et al., 2020). Low prevalence of help-seeking due to absence of distress matches the widely observed trend that older individuals frequently report symptoms of sexual problems but rarely express being concerned about them (Briken et al., 2020; Fischer and Træen, 2021; Graham et al., 2020; Hald et al., 2019; Richters et al., 2022). Consistently, the main reason for not seeking help reported in this study was not being bothered by the symptoms. It is possible that the problems were transient and manageable without intervention. Alternatively, older people often prioritize emotional intimacy and tenderness over physical sexual activity (Gore-Gorszewska and Ševčíková, 2023; Sandberg, 2013; Stahl et al., 2019). Adapting their sexual practices to focus on non-penetrative or non-physical intimacy may lessen the impact of sexual functioning problems and reduce the perceived need for professional support.

Besides the clear effect of distress, women were found to be less likely to report help-seeking for their sexual problems—a pattern also observed in the already mentioned four-country European survey on older adults (Hinchliff et al., 2020), among individuals aged 45 or over in the Natsal-3 (Hobbs et al., 2019), as well as in an analysis of sex-related queries posted by older adults on Czech professional counseling websites (Ševčíková et al., 2023). This consistent finding across studies is intriguing, given that women are generally more likely than men to visit healthcare professionals and to take on caregiving roles in the context of illness (Blackwell et al., 2014; Czech Statistical Office, 2023; Revenson et al., 2016). This may be explained by the well-recognized invisibility of female sexual dysfunction in both public discourse and medicine (Kaschak and Tiefer, 2014; Kleinplatz et al., 2020). The persistent disparity in attention given to women's healthcare compared to men's may discourage them from seeking help for their sexual problems (Khan et al., 2024). It is also possible that maintaining an active sex life is more important to aging men than women. A study by Gore-Gorszewska and Ševčíková (2023) points to a generational emphasis in Czechia on penetrative sex as the sole legitimate sexual practice in later life that may clash with the more intimacy-oriented expectations of older women. In this vein, the onset of sexual difficulties might function as an exit strategy for some women to withdraw from perceived marital obligations, including unsatisfying partnered sexuality (Gore-Gorszewska, 2021). Moreover, age differences typical for heterosexual couples could also help explain sex differences in help-seeking behavior. Younger women may be less motivated to address their own sexual difficulties if their older male partners are at greater risk of experiencing health issues that prevent them from having partnered sex (Hinchliff and Gott, 2004; Ševčíková and Sedláková, 2020). Hence, they may be more willing to reassess the importance of partnered sex in order not to put additional burdens on the partner.

It is worth mentioning that the logistic regression analysis largely failed to identify clear characteristics of no-help seekers. This group was very similar to those who sought help, which poses a challenge for effectively targeting no-help seekers and designing interventions. Other studies also struggle with considerable lack of association between selected—mainly demographic—characteristics and help-seeking behavior (Hinchliff et al., 2020; Hobbs et al., 2019), suggesting that more nuanced approach is necessary and future research should extend beyond the already considered sociodemographics and correlates of sexual life.

### Reasons for not seeking help

Almost a quarter of participants indicated that personal barriers, such as embarrassment, shame, reluctance to share, and difficulty in talking about sexual problems, stopped them from seeking professional help or counseling. Although such barriers are not exceptional and they have been observed in qualitative literature, they are usually not that common and framed within a doctor-patient interaction during consultation, that is fear of doctor's judgement, disapproval or dismissal of “irrelevant” concerns (Fileborn et al., 2017; Gore-Gorszewska, 2020; Schaller et al., 2020). Notably, in comparison with older adults from Norway, Denmark, Belgium and Portugal (Hinchliff et al., 2020), Czech women and men reported shame or embarrassment much more often. Possibly, cultural differences play a role in this discrepancy. This finding is particularly concerning given that the younger group (i.e., adults 50–59) was equally likely to report these personal/emotional barriers in help-seeking, suggesting a lack of generational progress in normalizing open communication about sex problems in this population.

Interestingly, systemic and institutional barriers were among the least reported reasons for not seeking professional help in this study, despite the costs and accessibility issues being recognized in literature (Maasoumi et al., 2023; Sever and Vowels, 2023). One explanation may be that personal barriers such as embarrassment, lack of knowledge, along with fear of doctor's disapproval may prevent individuals from even attempting to navigate the healthcare system thereby limiting critical evaluation of its usability.

## Limitations

The present study is limited by the sample size being insufficient to conduct separate analyses for late-midlife vs. older adults. These age cohorts are known to differ in how they approach and assess the severity of sexual difficulties (Lodge and Umberson, 2012). The tested model explained only 5% of the variance in seeking help. Future research should extend beyond the sociodemographic characteristics and correlates of sexual life and consider alternative explanatory factors (e.g., healthcare mistrust, health literacy). Moreover, several key constructs were measured using single items, following a widely adopted measurement approach (Briken et al., 2020; Mitchell et al., 2015). Although subjective health is considered one of the key factors influencing sexual functioning (Delamater, 2012) and help-seeking behavior in older women (Hinchliff et al., 2020), the logistic regression model did not take it into consideration due to respondents' health assessed only in the past four weeks. In order to ensure comparability with other population-based sex surveys—such as the GeSiD study—the Czech survey's battery of items omitted certain issues, such as vaginal dryness. This omission is notable, as vaginal dryness is among the most reported sexual difficulties in older women (Graham et al., 2020; Mitchell et al., 2013; Quinn-Nilas et al., 2018). Its exclusion may have contributed to an underestimation of the prevalence of female sexual difficulties and women's help-seeking in this study.

## Conclusion

This study found that nearly one in three Czechs aged 50–75 experienced a sexual problem in the past year, yet fewer than one in ten sought professional help. Women and those who perceived their problems as less distressing were especially unlikely to seek help. Importantly, help seekers and non-help seekers showed few differences in education, relationship status, sexual behavior, or attitudes toward sexual ageism, highlighting the challenge of identifying at-risk individuals through common demographic or attitudinal markers. While systemic barriers were infrequently reported, personal and emotional obstacles—such as shame, embarrassment, or minimizing the issue—were pervasive. These findings highlight the need for targeted, stigma-sensitive interventions even in secularized and atheistic Czechia that normalize help-seeking and improve communication around sexual health in later life. Such campaigns should specifically target the personal barriers that lead Czech individuals to downplay their sexual problems or avoid seeking professional help.

## Data Availability

The original contributions presented in the study are included in the article/Supplementary material, further inquiries can be directed to the corresponding author/s.
